# VISTA as a tumour-microenvironment checkpoint: spatial, metabolic and cellular control of antitumour immunity

**DOI:** 10.3389/fimmu.2026.1901272

**Published:** 2026-10-14

**Authors:** Kexun Li, Zilong Qian, Jianzhe Zhang, Jie Mao, Tengfei Ke

**Affiliations:** 1 Department of Thoracic Surgery, Sichuan Clinical Research Center for Cancer, Sichuan Cancer Hospital & Institute, Sichuan Cancer Center, Affiliated Cancer Hospital of University of Electronic Science and Technology of China (Sichuan Cancer Hospital), Chengdu, Sichuan, China; 2 Department of Thoracic Surgery I, Key Laboratory of Lung Cancer of Yunnan Province, Yunnan Cancer Hospital, The Third Affiliated Hospital of Kunming Medical University (Yunnan Cancer Hospital), Kunming, Yunnan, China; 3 Department of Radiology, The Third Affiliated Hospital of Kunming Medical University (Yunnan Cancer Hospital), Kunming, Yunnan, China

**Keywords:** cancer immunotherapy, immune checkpoint resistance, metabolic immune regulation, spatial immunology, tumour microenvironment, VISTA

## Abstract

V-domain immunoglobulin suppressor of T-cell activation (VISTA; also termed VSIR, PD-1H or B7-H5) is increasingly recognised as an immune checkpoint whose biological and clinical significance depends less on total expression than on the tumour microenvironment (TME) in which it operates. Rather than acting as a linear T-cell exhaustion receptor, VISTA integrates tumour chemistry, stromal architecture, innate and adaptive immune states, and therapy-induced feedback inhibition. It is expressed by tumour cells and multiple immune populations, including macrophages, myeloid-derived suppressor cells, dendritic cells, neutrophils, regulatory T cells and activated or quiescent T cells. VISTA can also function through pH-dependent and pH-independent ligand-receptor interactions. This review reframes VISTA as a TME-gated regulatory system. Low pH, hypoxia, transforming growth factor beta (TGF-β), histamine, aldehyde metabolism, Yes-associated protein (YAP)/TEA domain (TEAD) signalling, macrophage polarity, antigen-presenting-cell fitness and spatial immune exclusion can jointly determine whether VISTA marks immune resistance, immune activation under feedback restraint or tumour-cell-intrinsic evasion. This context helps explain the divergent prognostic associations of VISTA across pancreatic, gastric, colorectal, lung, breast, bladder, gynaecological, skin, brain, mesothelial and haematological malignancies. Therapeutically, VISTA should therefore not be treated as another universal immune-checkpoint target. More plausible strategies include pH-selective antibodies, ligand-axis disruption, local or nanoparticle delivery, targeted degradation, tumour-cell-intrinsic pathway inhibition and combinations that convert suppressive TME niches into antigen-presenting and cytotoxic niches. A TME-centred framework can make VISTA biology clinically interpretable and guide biomarker development, trial design and rational combination immunotherapy.

## Highlights

- VISTA should be interpreted as a tumour microenvironment (TME) checkpoint rather than a conventional single-axis T-cell checkpoint.
- The same VISTA signal can have different clinical meanings depending on pH, hypoxia, ligand usage, spatial compartment, tumour-cell expression and treatment history.
- VISTA integrates crosstalk among malignant cells, stromal architecture, macrophages, myeloid-derived suppressor cells (MDSCs), dendritic cells, regulatory T cells (Tregs), CD8+ T cells and natural killer (NK)-cell-relevant suppressive networks.
- Organ-specific evidence shows that VISTA can mark immune exclusion, adaptive resistance or feedback regulation in inflamed tumours; bulk VSIR expression is therefore insufficient as a biomarker.
- The most rational therapeutic approaches are TME-aware: pH-selective blockade, ligand-axis targeting, delivery systems, protein degradation and combinations that restore antigen presentation and cytotoxic-cell entry.

## 1 Introduction

The success of therapeutic blockade of programmed cell death protein 1 (PD-1), programmed death-ligand 1 (PD-L1) and cytotoxic T-lymphocyte-associated protein 4 (CTLA-4) created a powerful but incomplete model of cancer immunotherapy: inhibitory receptors silence T cells and therapeutic antibodies release the brake. VISTA challenges that model. It was first identified as a structurally unusual immunoglobulin superfamily molecule that suppresses T-cell responses and was subsequently shown to function as an immune checkpoint in human T cells and as a broad negative regulator of antitumour immunity (1–4). However, the subsequent literature has made clear that VISTA cannot be understood by asking only whether a cytotoxic T cell is exhausted. VISTA is distributed across multiple tumour-microenvironment (TME) compartments, including tumour cells, monocytes, macrophages, dendritic cells, neutrophils, myeloid-derived suppressor cells (MDSCs), regulatory T cells (Tregs) and activated or quiescent T cells (5–11).

A TME-centred interpretation is therefore more informative than a lineage-restricted one. Tumours are not simply masses of malignant cells surrounded by passive immune infiltrates; they are ecosystems shaped by acidity, hypoxia, fibrosis, nutrient stress, cytokines, chemokines, antigen availability, vascular dysfunction and therapy-imposed selection. VISTA sits at several of these interfaces. It restrains T-cell activation, contributes to immune quiescence and modulates myeloid inflammation (5–11, 35). VISTA can also cooperate with metabolic and inflammatory programmes (27–32) and can be expressed by malignant cells themselves (33, 34, 45–52). In this sense, VISTA is not merely present in the TME; it can help define whether the local immune environment is permissive or hostile to productive antitumour immunity.

This reframing also resolves an important contradiction. Some studies associate VISTA with immune suppression and poor outcome, whereas others link it to abundant immune infiltration or favourable prognosis (13–18). Such divergence is not necessarily evidence that the field is confused. This provides evidence that VISTA is context-dependent. VISTA expressed on secreted phosphoprotein 1 (SPP1)-like macrophages in pancreatic cancer (40–44), on VISTA-high immune cells in gastric cancer (61) or on Yes-associated protein (YAP)-active colorectal cancer cells (33) is unlikely to have the same biological meaning as VISTA enrichment within urothelial immune-inactivation niches (79, 80) or VISTA detected in lymphocyte-infiltrated colorectal, ovarian or cutaneous squamous-cell-carcinoma lesions (47, 63, 87).

Conventional immunohistochemistry may identify positivity but cannot determine the ligand partner, pH state, local hypoxia or distance from cytotoxic cells, while bulk sequencing cannot preserve the spatial neighbourhood in which a VISTA signal becomes functional (13–18, 65, 81). The logic of the review is therefore organised around five linked questions. First, molecular structure and ligand usage dictate what VISTA can do. Secondly, tumour chemistry and inflammatory signals regulate whether those capabilities are engaged. Thirdly, cellular compartments translate VISTA activity into altered antigen presentation, suppressive differentiation or cytotoxic failure. Fourthly, spatial organisation determines whether these effects reach tumour-reactive lymphocytes. Finally, therapeutic pressure can either reveal VISTA dependence or induce compensatory VISTA-associated resistance. A tumour with high VISTA, hypoxia, MDSC accumulation and scarce intratumoural T cells may require metabolic or stromal reconditioning before checkpoint blockade can work (19–23, 27, 28), whereas tumours with malignant-cell VISTA driven by YAP, nuclear factor kappa B (NF-κB) or protein-stability pathways may require tumour-cell-intrinsic intervention or degradation strategies (31, 33, 34, 105, 106).

## 2 Molecular wiring and environmental gating of VISTA

VISTA is a type I transmembrane protein with a single immunoglobulin variable (IgV)-like extracellular domain and an intracellular tail that lacks canonical immunoreceptor tyrosine-based inhibitory motif (ITIM), immunoreceptor tyrosine-based activation motif (ITAM) or immunoreceptor tyrosine-based switch motif (ITSM) sequences. Its cytoplasmic region nevertheless contains conserved proline-rich and kinase-associated features, implying non-classical signalling and explaining why VISTA behaves neither as a simple PD-1 substitute nor as a purely passive ligand (5, 7–11). This structural ambiguity matches its biological versatility: VISTA may act as a ligand on myeloid or tumour cells, as a receptor-like molecule in T cells, and as a tumour-cell-intrinsic regulator in selected cancers (**Figure 1**).

**Figure 1:**
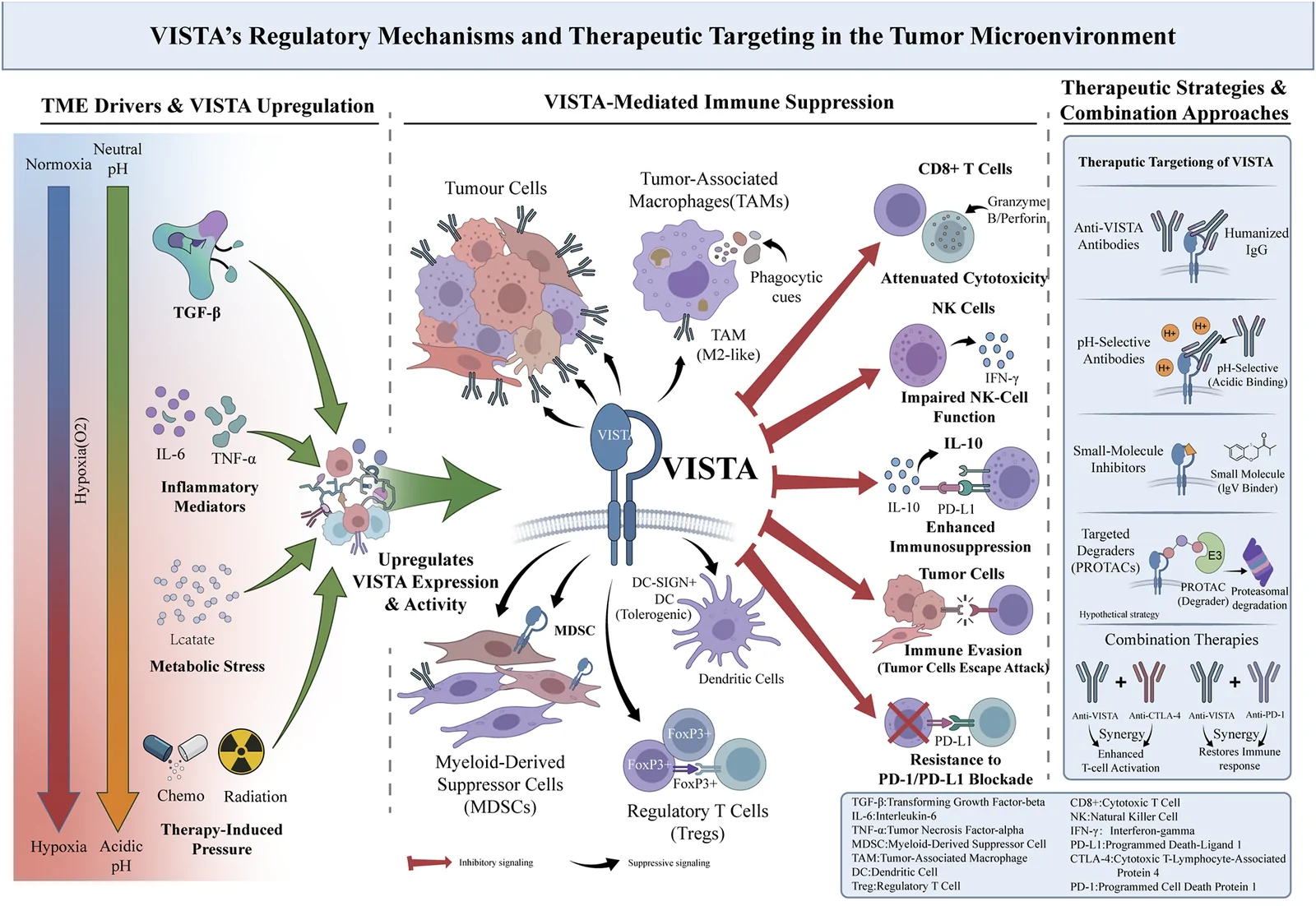
The visual abstract of VISTA and the logic of context-dependent immune resistance in cancer.

### 2.1 Physiological and non-malignant functions of VISTA

Before considering cancer-associated roles, VISTA should be placed within its physiological function as a regulator of immune homeostasis. In non-malignant settings, VISTA contributes to naive T-cell quiescence and peripheral tolerance and restrains inflammatory activation across monocytes, macrophages, neutrophils and microglia (5–7, 53, 54). These functions limit inappropriate cytokine amplification and tissue injury rather than primarily mediating tumour immune escape (53, 54). Cancer-associated VISTA biology can therefore be viewed, in part, as the co-option or spatial reprogramming of a physiological tolerance pathway within the TME. This distinction is therapeutically relevant because complete systemic inhibition may disrupt protective immune homeostasis (6, 7, 53, 54).

### 2.2 Ligand landscape and tumour-microenvironmental gating

Within cancer, the most important environmental principle is pH gating. VISTA engages P-selectin glycoprotein ligand-1 (PSGL-1) preferentially under acidic conditions through a histidine-rich interface, allowing this axis to become most active in glycolytic, poorly perfused and metabolically stressed tumour regions (19–21). This finding moved VISTA biology from a generic checkpoint concept to a TME-chemistry concept. It also explains why systemic expression does not automatically predict function and why pH-selective antibodies were designed to recognise active VISTA preferentially in acidic tumour niches rather than in normal blood or tissue (22, 23).

The VISTA ligand landscape can be organised into several functional classes. First, the acidic-pH-gated PSGL-1/VISTA axis links tumour chemistry directly to T-cell suppression (19–23). Second, pH-independent cell-surface interactions include V-set and immunoglobulin domain-containing 3 (VSIG3; also known as immunoglobulin superfamily member 11, IGSF11), which can suppress human T-cell function through VISTA (24, 25), and leucine-rich repeats and immunoglobulin-like domains protein 1 (LRIG1), a VISTA-binding inhibitory receptor on activated tumour-specific CD8+ T cells (26). Third, additional context-specific or proposed partners broaden the landscape. Galectin-9 has been reported to bind VISTA in acute myeloid leukaemia and to contribute to impaired T-cell cytotoxicity (91), whereas matrix metalloproteinase 13 (MMP-13) has been linked to VISTA/PD-1H receptor activity on osteoclasts in multiple myeloma bone disease (53, 91). These less universal interactions should be interpreted as context-specific axes rather than assumed to operate across all tumours. Together, the ligand landscape suggests that VISTA functions as a molecular switchboard in which pH, ligand availability, cellular compartment and activation state determine biological output.

Other TME factors regulate VISTA abundance and function. Hypoxia and hypoxia-inducible factor 1 (HIF-1) signalling can induce VISTA in suppressive myeloid contexts (27, 28). Transforming growth factor beta (TGF-β) can modulate VISTA-related intracellular and extracellular activities (32), whereas aldehyde metabolism can upregulate VISTA through a nucleotide-binding oligomerisation domain (NOD)/NF-κB axis (31). Histamine signalling through histamine H1 receptor (H1R) can polarise macrophages toward immunosuppressive states with increased VISTA (29, 30). In malignant cells, YAP/TEA domain transcription factor 4 (TEAD4)/specificity protein 1 (SP1) signalling or heat shock protein 90 alpha family class B member 1 (HSP90AB1)/NF-κB pathways can promote tumour-cell-intrinsic VISTA programmes in colorectal or ovarian cancer (33, 34).

This regulatory plasticity is a key difference between VISTA and a simple therapeutic antigen. A tumour can generate VISTA activity by increasing receptor abundance, shifting pH to favour a specific ligand interaction (19–23), positioning VISTA-positive antigen-presenting cells (APCs) next to tumour-reactive T cells (4, 35–37), inducing tumour-cell expression through oncogenic transcription factors (33, 34), or altering protein stability (105, 106). Conversely, the same tumour may lose VISTA dependence if antigen priming is absent, if T cells cannot enter the lesion, or if a different suppressive axis dominates. The therapeutic implication is that VISTA should be assayed together with the environmental signal that makes it functional. pH-related signatures, hypoxia markers, TGF-β activity, H1R signalling, aldehyde dehydrogenase 2 (ALDH2) activity, YAP/TEAD activation, NF-κB signalling and ligand proximity are not secondary details; they are part of the checkpoint (19–34).

## 3 Cellular circuits: VISTA as a coordinator of TME crosstalk

The TME-level action of VISTA can be viewed as a circuit rather than a cell-autonomous phenotype. In APCs, VISTA restrains inflammatory maturation and antigen presentation, thereby limiting the priming and maintenance of cytotoxic T-cell responses (4, 35–37). In MDSCs, VISTA supports suppressive differentiation, metabolic fitness and T-cell inhibition, with signal transducer and activator of transcription 3 (STAT3) and polyamine-dependent mechanisms providing a direct bridge between immune metabolism and checkpoint regulation (27, 28). In dendritic-cell-rich settings, VISTA blockade or knockdown can increase activation markers and promote T-cell entry, but the magnitude of this effect depends on whether antigen and inflammatory signals are also available (4, 36, 37).

Macrophages are a prominent TME interface for VISTA, but the point is not that VISTA belongs only to macrophages. Rather, macrophages illustrate how VISTA converts a local environmental state into a global immune consequence. T-cell immunoglobulin and mucin-domain containing-3 (TIM-3)+VISTA+ tumour-associated macrophages (TAMs) are conserved across resistant tumours and are enriched in low-immunogenicity, T-cell-sparse and interleukin-4 (IL-4)-rich contexts (38, 39). Their blockade, especially when paired with immunogenic cell death-inducing therapy, can reprogramme macrophages from tumour-supportive states toward inflammatory antitumour states (38, 39). In pancreatic ductal adenocarcinoma (PDAC), recent evidence further connects VISTA to the C-X-C motif chemokine ligand 9 (CXCL9):SPP1 axis of macrophage polarity, antigen cross-presentation and recruitment of C-X-C motif chemokine receptor 3 (CXCR3)+ cytotoxic T cells (40–44). These findings make VISTA a local organiser of macrophage-T-cell geography within tumours.

T cells experience VISTA at several phases of the antitumour response. A VISTA-immunoglobulin fusion protein (VISTA-Ig) suppresses proliferation and cytokine release, can support forkhead box P3 (FOXP3)+ regulatory skewing, and maintains quiescence-like states (1, 2, 5, 7). In established tumours, VISTA-associated functional consequences include reduced CD8+ T-cell infiltration, lower interferon gamma (IFN-γ) and granzyme programmes, increased Treg activity and failure of antigen-specific responses to translate into tumour control (4, 21, 26, 31, 38).

Tumour-cell VISTA adds another layer. In gynaecological tumours, tumour-cell VISTA can suppress T-cell proliferation, reduce IFN-γ and tumour necrosis factor alpha (TNF-α), lower local CD8+ T-cell abundance and increase MDSC accumulation (45–51). In colorectal cancer, YAP/TEAD4/SP1-induced VISTA provides a tumour-cell-intrinsic route to immunosuppression (33), whereas one study suggests that intracellular VISTA motifs may, in some settings, restrain epidermal growth factor receptor (EGFR) trafficking and tumour growth (52). Together, these observations show that VISTA can be an extracellular checkpoint, an intracellular signalling participant, a tumour-cell immune-evasion module or, in defined contexts, part of growth-restraining biology.

The same circuitry probably affects natural killer (NK) cells and other innate lymphoid outputs indirectly. Single-cell studies of tumour-associated NK-cell dysfunction and broader TME atlases emphasise that cytotoxic failure often arises from suppressive myeloid and stromal networks, not only from intrinsic NK-cell defects (15, 53–56). Because VISTA alters antigen presentation, macrophage cytokines, Treg support and chemokine structure, it may influence NK-cell access and function even when NK cells are not the primary VISTA-expressing compartment (15, 53–56). Thus, a TME-centred model treats VISTA as a regulator of communication among compartments, not as a marker locked to one lineage.

Regulatory T cells, neutrophils and tolerogenic dendritic cells are equally important in this communication map. VISTA expression in Tregs can reinforce local suppression (5–11), whereas VISTA-positive dendritic cells may fail to generate the costimulatory and chemokine signals needed for durable cytotoxic immunity (4, 35–37). Neutrophil-rich TMEs, which are often hypoxic and protease-rich, may further amplify tissue-repair and tolerance programmes (53, 54). These compartments are not always the best characterised in VISTA studies, but their inclusion prevents an overly narrow model. The relevant output is a coordinated TME state characterised by poor antigen presentation, reduced effector cytokines, suppressive cytokine dominance, altered chemokine gradients, T-cell quiescence, spatial exclusion and adaptive checkpoint redundancy.

## 4 Spatial and organ-specific heterogeneity

High VSIR or VISTA expression is associated with immune infiltration, co-inhibitory molecules, tumour mutational features and survival in multiple datasets, but the direction of association varies by tumour type and analytic method (13–18). Bulk transcriptomic measurements can be dominated by immune-cell abundance, whereas protein immunohistochemistry may separate tumour, stromal, macrophage, lymphocyte and margin-specific signals (15, 65).

Gastrointestinal cancers show the full range of TME meanings. In PDAC, VISTA is repeatedly connected to an immunosuppressive, macrophage-rich and T-cell-resistant TME; VISTA blockade or deficiency can alter TAM polarity, increase cross-presentation and improve cytotoxic T-cell recruitment (40–44, 57). In gastric cancer, VISTA-high tumours have recently been characterised as immunosuppressive immune-microenvironment landscapes, reinforcing the idea that VISTA can identify a TME state rather than a single cell population (58–61). In colorectal cancer, by contrast, VISTA can correlate with favourable prognosis in some cohorts (63), yet APC-truncated or YAP-active contexts may reveal VISTA-dependent immune escape and therapeutic vulnerability (33, 62, 64).

Lung cancer also illustrates why VISTA cannot be judged by expression alone. Spatial quantitative studies of non-small cell lung cancer (NSCLC) show VISTA protein in stromal and immune compartments and sometimes in tumour cells, with correlations to PD-1, PD-L1, CD8+ T cells and CD68+ macrophages (65, 66). Experimental lung models suggest that VISTA/PD-L1-targeting small molecules or VISTA blockade can enhance CD8+ T-cell infiltration, reduce suppressive myeloid populations and complement antigen-specific vaccination (67, 68). Meanwhile, anti-PD-L1 therapy can reshape monocyte states and induce alternative checkpoint molecules, including VISTA, suggesting a role in adaptive resistance rather than only baseline suppression (69).

In breast cancer, VISTA has been linked to radiation-immunotherapy responses, epithelial-mesenchymal transition (EMT)-like programmes, PD-1-related immune structure, macrophage polarisation and, in some triple-negative cohorts, favourable immune-cell-associated prognosis (70–78). In muscle-invasive bladder cancer, VISTA+ immune-cell infiltration is associated with worse survival and weaker chemotherapy benefit but may predict benefit from PD-L1 blockade, indicating a suppressed yet therapeutically targetable immune-inflamed state (79, 80). Head-and-neck cancer analyses have implicated the STAT3-VSIR axis and spatial checkpoint patterns in immunotherapy response (81–83). In ovarian and endometrial cancers, VISTA can be expressed by tumour and immune cells and can mark either immunosuppression or an immune-active context, depending on coexpression with CD8, CTLA-4, PD-1 and tumour-cell programmes (34, 46–51).

Other tumour types underline the need for context. Soft-tissue sarcomas (84), uveal melanoma (85, 86), cutaneous squamous cell carcinoma (87), glioma (54–56), mesothelioma (88, 89) and haematological malignancies (90, 91) all show VISTA expression with variable clinical interpretation. Mesothelioma is relevant because VISTA can be highly expressed and potentially targetable (88, 89), whereas cutaneous squamous cell carcinoma may show correlation with T-cell infiltration and activation (87). These examples argue against a universal ‘VISTA-high equals VISTA-dependent’ rule. Instead, they support a decision model in which VISTA expression is integrated with immune-cell type, tumour-cell state, stromal compartment, T-cell location, pH-related chemistry and prior treatment (15, 17, 18).

Spatial heterogeneity also explains why VISTA may have different meanings within one patient. A metastatic lesion can contain acidic necrotic cores, vascularised immune-active margins, fibrotic exclusion zones and tertiary lymphoid structures, each with different VISTA biology (15, 56, 65, 81). A biopsy taken from the edge of a lesion may therefore report an inflamed feedback state, whereas a core sample may report immune exclusion and macrophage-driven suppression. Serial sampling adds another dimension because chemotherapy, radiation, anti-angiogenic therapy and immune checkpoint blockade can alter antigen release, vascular permeability, macrophage differentiation and compensatory checkpoint expression (69, 71, 79, 81). VISTA should consequently be interpreted as a dynamic TME feature.

## 5 Therapeutic translation: remodel the TME, not simply block a receptor

If VISTA is a TME-gated checkpoint, therapy should aim to remodel the TME rather than only occupy a surface molecule. Antibody development illustrates this point. Non-depleting antibodies such as HMBD-002 were designed to block function without necessarily eliminating VISTA+ cells (92, 93), whereas pH-selective antibodies such as SNS-101 exploit the acidic-state biology of VISTA to widen the therapeutic window and reduce risks associated with systemic VISTA engagement (22, 23). Fragment crystallisable (Fc) engineering also matters because VISTA is expressed on immune subsets whose depletion or cross-linking could be beneficial or harmful depending on context (94).

Small molecules and peptides represent a second strategy: disrupt microenvironmentally relevant ligand-receptor interactions or, for selected chemical scaffolds, modulate VISTA protein abundance. Peptide inhibitors and chidamide-based repositioning approaches specifically target the acidic-pH-dependent VISTA/PSGL-1 interaction (21, 95). Methoxy-pyrimidine, benzimidazole and bifunctional PD-L1/VISTA compounds show that VISTA can be approached with orally oriented or degradation-capable chemistry, although much of this work remains preclinical (96–104). Recent VISTA destruction studies are conceptually important because they shift the field from blockade to protein regulation: VISTA can be degraded, destabilised or regulated through E3 ligase and deubiquitinase biology (105, 106). This is particularly attractive for tumour-cell-intrinsic VISTA or intracellular VISTA functions that antibodies may not access.

Delivery and local activation strategies further fit the TME framework. VISTA-specific small interfering RNA (siRNA) and Toll-like receptor 9 (TLR9) agonist co-loaded nanoparticles can increase antitumour immunity while limiting systemic cytokine release (37). VISTA-targeted near-infrared photoimmunotherapy can locally eliminate VISTA+ suppressive immune cells and enhance host immunity (107). VISTA antibody-loaded sonodynamic nanoparticles in pancreatic cancer combine physical TME modification, immunogenic cell death, stromal penetration and checkpoint targeting (108). These modalities are attractive because they treat VISTA as part of a spatial problem: the relevant cells may be clustered in hypoxic, fibrotic or immune-excluded regions that conventional antibodies reach poorly.

The most plausible clinical future is combination therapy. VISTA targeting may be paired with PD-1/PD-L1 blockade when adaptive resistance is documented (69, 92, 93), with CTLA-4 or TIM-3 targeting in macrophage-rich resistant niches (36, 38), with vaccines when antigen-specific T-cell priming is inadequate (36, 68), or with radiotherapy, chemotherapy, local delivery or stromal-modulating strategies when immunogenic cell death or tumour entry is limiting (71, 107, 108). However, combinations should not be selected solely because a pathway is of current therapeutic interest. They should be chosen to repair a defined TME failure, such as insufficient antigen presentation, acidic PSGL-1 signalling, suppressive macrophage polarity, Treg dominance, exclusion from tumour nests, tumour-cell-intrinsic VISTA induction or therapy-induced compensatory checkpoint expression (19–34, 38–44, 65–69, 79–83).

This principle also clarifies why some VISTA programmes may fail. An antibody that blocks VISTA cannot compensate for absent tumour antigenicity, lack of dendritic-cell priming, irreversible stromal exclusion or dominant non-VISTA suppressive pathways. A small molecule designed to destabilise or degrade VISTA may have limited effect if drug penetration is poor in the relevant niche (97, 105, 106). A pH-selective antibody may be ideal for acidic lesions but less suited to neutral-pH VSIG3- or LRIG1-dominant interactions (19–26). The best translational designs will therefore begin with a TME diagnosis and then choose the modality: antibody blockade, pH-selective blockade, ligand-axis disruption, protein degradation, RNA delivery, photoimmunotherapy, sonodynamic therapy or combination priming (19–26, 92–108).

## 6 Biomarker and trial design principles for a TME-centred VISTA programme

A useful VISTA biomarker must answer four questions. First, which cell expresses VISTA? Second, where is that cell located relative to malignant cells, vessels, stroma, necrosis, tertiary lymphoid structures and cytotoxic T cells? Third, which ligand axis and environmental gate are plausible in that region? Fourth, what therapy has already reshaped the TME? Without these answers, a VISTA-high biomarker is too crude. Multiplex immunofluorescence, spatial transcriptomics, single-cell ribonucleic acid (RNA) sequencing and digital pathology can resolve cell identity and neighbourhood structure (15, 56, 65, 81), while ex vivo tumour cultures and serial pharmacodynamic biopsies can test whether treatment changes the predicted VISTA circuit (107, 108) (**Figure 2**).

**Figure 2:**
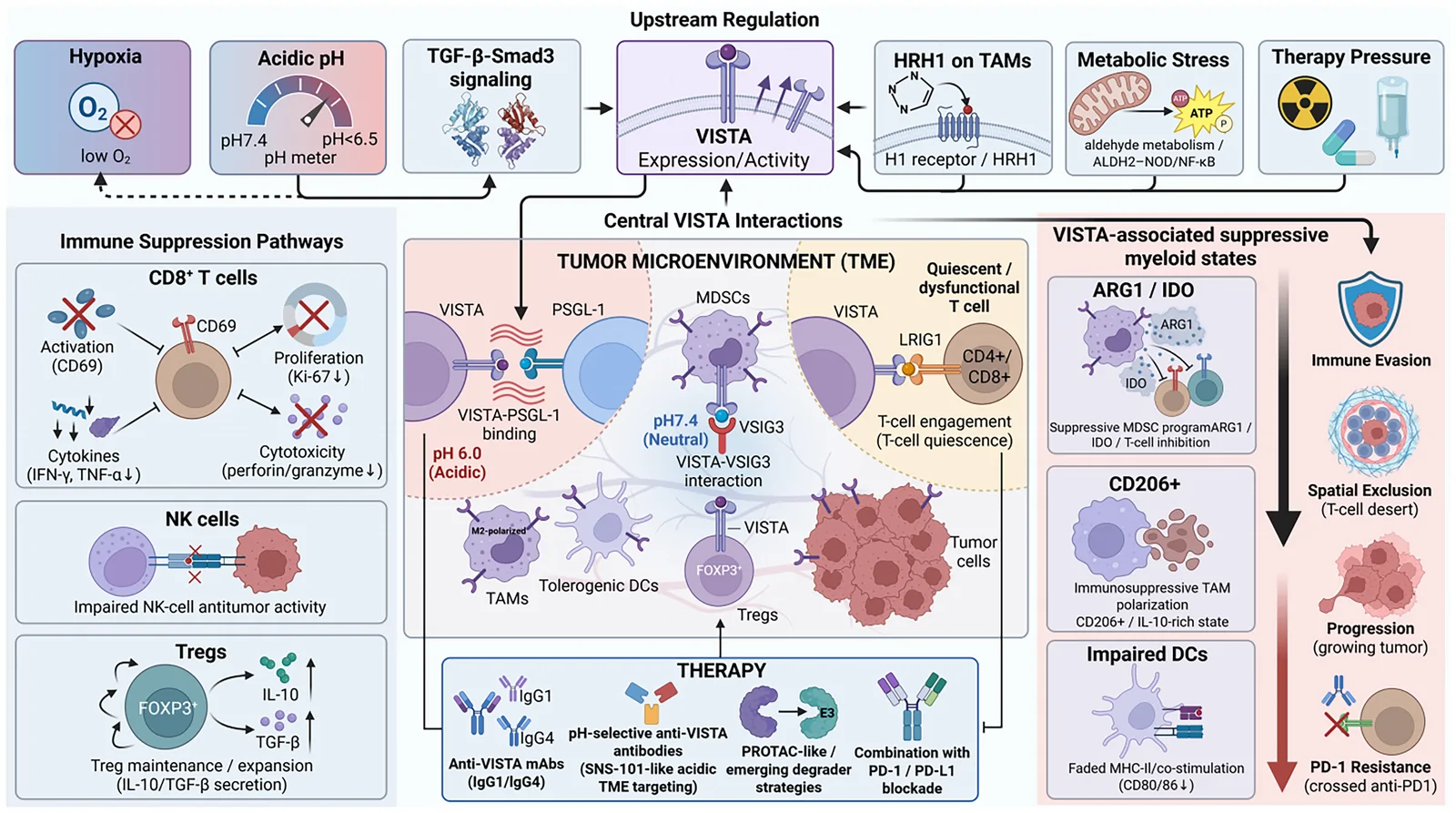
VISTA mechanism in single-checkpoint and network immunotherapy.

Trial endpoints should test mechanism. In PDAC, a VISTA programme should measure whether SPP1-like TAM states shift toward CXCL9-rich antigen-presenting states, whether CXCR3+ CD8+ T cells enter tumour nests and whether exhaustion-like terminal states fall (44). In gastric cancer, studies should test whether VISTA-high immune-suppressed landscapes can be converted into antigen-presenting and cytotoxic landscapes (61). In bladder cancer, the relevant question is whether VISTA+ immune inactivation predicts PD-L1 benefit or identifies patients needing dual blockade (79). In colorectal or ovarian cancer, tumour-cell VISTA programmes should be measured alongside YAP/TEAD, NF-κB, EMT and CD8 exclusion (33, 34).

Safety must be considered with equal seriousness. VISTA participates in immune homeostasis in monocytes, macrophages, neutrophils, microglia and resting T cells (6, 7, 53, 54). Systemic and complete pathway inhibition may therefore carry risks of inflammatory toxicity, cytokine release or disruption of tissue tolerance (6, 7, 53, 54). A TME-centred programme would favour tumour-selective exposure, conditional activity, careful dose escalation and pharmacodynamic confirmation over maximal systemic blockade. The design question is not only how to inhibit VISTA, but how to inhibit the pathogenic VISTA circuit while preserving protective immune homeostasis.

The field also needs better negative-study interpretation. If a VISTA antibody fails without changing macrophage polarity (38–44), pH-restricted ligand engagement (19–23), antigen presentation (35–37) or cytotoxic-cell entry (44, 65, 81), the trial has not truly tested the TME hypothesis. Conversely, a therapy that reduces VISTA protein (105, 106) but leaves fibrotic exclusion, hypoxia and absent antigen priming untouched may also fail despite target engagement. Mechanistic endpoints should therefore be built into early trials rather than added after disappointing efficacy. VISTA research will mature when clinical studies are designed around microenvironmental states instead of expression alone.

A practical biomarker panel might therefore include four layers. The first layer is cellular: VISTA in tumour cells, macrophages, MDSCs, dendritic cells, Tregs and activated CD8+ T cells (5–11, 33, 34, 40–51). The second is spatial: distance between VISTA-positive cells and tumour nests, CD8+ cells, vessels, necrosis, collagen-rich stroma and lymphoid aggregates (15, 56, 65, 81). The third is environmental: pH or glycolysis proxies, hypoxia, TGF-β, histamine, ALDH2 or YAP/NF-κB activity (19–34). The fourth is treatment history: baseline, after chemotherapy or radiotherapy, after PD-1/PD-L1 blockade, after CTLA-4 blockade or during neoadjuvant therapy (69, 71, 79, 81). A biomarker built from these layers would be more complex than PD-L1 immunohistochemistry, but it would match the biology more closely.

Instead of stating that a tumour is VISTA-positive, reports should specify whether VISTA is tumour-cell predominant, immune-cell predominant or spatially mixed; whether VISTA-positive cells are intratumoural, stromal, perivascular, necrosis-adjacent or margin-restricted; and whether VISTA co-localised with PSGL-1, VSIG3, LRIG1 or other context-specific partners (19–26, 53, 91). Reports should also indicate whether VISTA positivity coincides with hypoxia, glycolysis, fibroblast barriers or immune-cell exclusion (19–34, 65, 81). This language may seem demanding, but modern TME biology increasingly treats immune state as a spatial and functional architecture (15, 65, 81). VISTA deserves the same standard. Without it, the field will keep producing apparently contradictory prognostic studies; with it, those contradictions become testable hypotheses about which compartment dominates a given lesion (13–18).

## 7 A practical TME model

In a hypoxic, myeloid-suppressive tumour, VISTA is likely to function through PSGL-1 engagement, MDSC fitness, TAM polarisation and impaired antigen presentation (19–23, 27, 28, 38–44). In an inflamed tumour with abundant T cells, VISTA may reflect feedback restraint and require combination with PD-1 or CTLA-4 blockade rather than single-agent targeting (12, 36, 61, 79). In tumour-cell VISTA states, transcriptional or protein-stability programmes such as YAP/TEAD, NF-κB, HSP90AB1, tripartite motif-containing 25 (TRIM25) and ubiquitin-specific peptidase 2 (USP2) become part of the therapeutic hypothesis (31, 33, 34, 105, 106). In spatially excluded tumours, local delivery, stromal modulation or immunogenic cell-death strategies may be needed before VISTA blockade can expose tumour cells to cytotoxic immunity (**Table 1**) (37, 107, 108).

**Table 1:** TME determinants that change the interpretation of VISTA.

TME determinant	How it shapes VISTA biology	Preferred biomarker readout	Therapeutic implication
Acidic pH and glycolytic stress	Favours PSGL-1/VISTA engagement and T-cell suppression in metabolically stressed niches	Spatial pH/hypoxia signatures plus VISTA and PSGL-1 proximity	pH-selective antibodies or ligand-axis disruptors
Hypoxia, TGF-β and aldehyde metabolism	Induce or modulate VISTA expression through HIF-1, TGF-β and NOD/NF-κB-related programmes	HIF-1/TGF-β/ALDH2 signatures with cell-type resolved VISTA	Combine VISTA targeting with metabolic or inflammatory reprogramming
Macrophage and MDSC states	Convert VISTA into a suppressive circuit affecting antigen presentation, cytokines and CD8+ T-cell entry	SPP1:CXCL9 ratio, STAT3/polyamine programmes, MDSC suppressive assays	Target VISTA with macrophage reprogramming, immunogenic cell death-inducing therapy or PD-1 blockade
Tumour-cell-intrinsic VISTA	Suppresses T-cell function or reflects oncogenic transcriptional/protein-stability programmes	Tumour-cell VISTA by spatial pathology plus YAP/TEAD, NF-κB or TRIM25-USP2 markers	Use blockade, degradation or upstream pathway inhibition when tumour-cell expression dominates
Immune-inflamed feedback restraint	VISTA may mark immune activation under inhibitory feedback rather than primary immune exclusion	CD8/Treg/APC spatial maps and co-checkpoint panels	Combine with PD-1/PD-L1, CTLA-4 or vaccine strategies rather than single-agent targeting
Fibrosis and spatial exclusion	Limits drug penetration and separates cytotoxic cells from tumour nests	Tumour-stroma maps, vessel density, immune-cell distance metrics	Local delivery, sonodynamic/photoimmunotherapy or stromal modulation before or with VISTA targeting

This model does not diminish the importance of any individual compartment. It places each compartment within a larger ecological sequence: tumour stress and stromal architecture set the chemical niche (19–34); VISTA-positive tumour or immune cells impose quiescence and antigen-presentation failure (1–11, 35–37); suppressive macrophages, MDSCs and Tregs maintain exclusion or dysfunction (27, 28, 38–44); cytotoxic T cells and NK-associated responses fail to reach or persist in malignant nests (15, 53–56, 65); and treatment pressure can select compensatory checkpoint and cytokine programmes (69, 79–83). The therapeutic goal is to interrupt the sequence at the point that dominates a given patient’s tumour.

A pancreatic tumour with dense stroma, SPP1-rich macrophages and few intratumoural CD8+ T cells (40–44) raises a different VISTA hypothesis from a gastric tumour with VISTA-high immune infiltration (61), a colorectal tumour with tumour-cell YAP activity (33), or a bladder tumour with inflamed but exhausted immune-cell aggregates (79). The same immunohistochemical score may therefore lead to different therapeutic decisions. This is not an inconvenience but a useful precision-medicine principle: VISTA can help identify which part of the immune ecosystem is failing.

The model is also intentionally falsifiable. If VISTA is driving an acidic PSGL-1 state, then disruption should increase local T-cell activation in acidic regions and pharmacodynamic markers should differ from neutral regions (19–23). If VISTA is driving a macrophage-polarity state, then CXCL9, antigen presentation and CD8+ proximity should change (38–44). If tumour-cell VISTA is dominant, then tumour-cell expression, oncogenic transcription factors and T-cell suppression in co-culture or organotypic models should change (33, 34). If VISTA is merely a bystander marker of immune infiltration, then blockade may not alter the TME despite high expression (13–18). Such discriminating hypotheses are essential for moving beyond descriptive biomarker studies.

## 8 Conclusion

VISTA should be interpreted as a context-dependent regulator of the tumour microenvironment rather than as a uniformly favourable or unfavourable checkpoint. Its biological effect depends on local acidity, hypoxia, inflammatory signalling, stromal organisation, antigen-presenting-cell function, myeloid suppression and T-cell state. This explains why VISTA may correlate with poor outcome in some tumours but with immune infiltration or favourable prognosis in others. Future studies should therefore move beyond bulk expression and define the dominant VISTA-expressing cell type, ligand axis, spatial niche and treatment-induced change. Clinically useful VISTA targeting will require evidence that pathway modulation restores antigen presentation, improves cytotoxic-cell positioning and produces measurable pharmacodynamic effects in patient tumours.
